# The next hype in social media advertising: Examining virtual influencers’ brand endorsement effectiveness

**DOI:** 10.3389/fpsyg.2023.1089051

**Published:** 2023-02-24

**Authors:** Eunjin (Anna) Kim, Donggyu Kim, Zihang E, Heather Shoenberger

**Affiliations:** ^1^Annenberg School for Communication and Journalism, University of Southern California, Los Angeles, CA, United States; ^2^Donald P. Bellisario College of Communication, Penn State University, University Park, PA, United States

**Keywords:** human-like virtual influencers, animated virtual influencers, sponsorship disclosure, message credibility, message attitude, brand endorsement

## Abstract

Virtual influencers are gaining prominence as a way of attracting people’s attention on social media, but limited research has been conducted on this subject. In this research, we explore the effects of human-like virtual influencers (HVIs) vs. anime-like virtual influencers (AVIs) and sponsorship disclosure on message credibility perception and message attitudes. Conducted with a 2 (virtual influencer type: HVI vs. AVI) x 2 (sponsorship disclosure: absent vs. present) between-subjects experiment, our findings suggest that HVI endorsements produce greater perception of message credibility and message attitudes than AVI endorsements, but the superior effect of HVIs (vs. AVIs) vanishes when sponsorship is disclosed. The results also show that message credibility plays a significant mediating role only when sponsorship is not disclosed. We believe our research offers interesting insights to both researchers and practitioners on the topic of virtual influencers.

## 1. Introduction

Social media influencers (SMIs) have been incredibly effective in the advertising arena, and virtual influencers (VIs) are an intriguing counterpart with the potential to harness the positives of human influencers with additional control over content and expression. In fact, brands have been increasingly partnering with VIs (Conti et al., 2022). According to a recent industry report, 58% of the respondents were following at least one VI and 35% of them said that they had purchased a product promoted by a VI (The Influencer Marketing Factory, 2022). The global VI industry is growing so fast, and its market value in China alone is expected to reach $42.6 billion by 2030 (Jing Daily, 2022).

VIs are artificial in nature while displaying the same type of content as real human influencers (Stein et al., 2022). According to Thomas and Fowler (2021, p. 12), they are “digitally created artificial humans who use algorithms and software to perform tasks like humans.” Research has shown that VIs can be created with personas that can connect digital audiences in more productive and meaningful ways than human influencers can (Arsenyan and Mirowska, 2021).

VIs exist on a spectrum of levels of anthropomorphism in terms of their appearance from quite obviously anime-like to almost indistinguishable from a human. Despite the wide range of VIs (e.g., anime-like to human-like), limited studies (Choudhry et al., 2022; Conti et al., 2022; Liu and Lee, 2022; Sands et al., 2022; Stein et al., 2022) have examined people’s perception about different types of VIs. For example, Choudhry et al. (2022) conducted interviews about how Instagram users respond to different types of VIs (i.e., human-like, anime-like, and animal-like) and found that human-like VIs were perceived to be more attractive. Yet, since the usage of VIs for brand endorsement is still relatively new, little is known about the implications and the effects VIs have on brands. As VIs are increasing in demand for collaboration with brands (Hype Auditor, 2021), more empirical research is needed to fully understand what role VIs play in advertising and what impact the difference between human-like and anime-like VIs has on brand promotion.

Specifically, informed by Computers Are Social Actors (CASA, Gambino et al., 2020) paradigm, we propose that HVIs (vs. AVIs) will produce a greater perception of message credibility, which, in turn, generates more positive message attitudes. Current FTC regulations require that SMIs disclose their material connection with a sponsor when it comes to brand-sponsored posts, and there is speculation that VIs will soon be required to abide by the same rules (Masteralexis et al., 2021). Thus, we look at the variable of sponsorship disclosure and propose it as an important boundary condition for the effectiveness of HVI (vs. AVI) endorsements.

We believe this research contributes to the growing body of knowledge on the effectiveness of influencer endorsement and sponsorship disclosure by extending the literature to the virtual influencer phenomenon. This research also provides practitioners with valuable insights into how to utilize VIs as a marketing tool. Overall, we expect our research will spark more interest in this important topic from both scholars and practitioners.

## 2. Literature review

### 2.1. Virtual influencers

VIs are one of the latest trends in influencer marketing campaigns (Kadekova and Holienčinova, 2018). They have public identities and storylines, much like human influencers, leading to increased engagement between users and influencers in the digital realm (Hanus and Fox, 2015). Studies have found that consumers are becoming increasingly familiar with virtual agents in brand interaction contexts (Sands et al., 2020) and view VIs favorably (Thomas and Fowler, 2021), showing substantial marketing advantages. Lately, brands have started to use VIs to promote their products, and many have achieved unexpected success. With over 3 million Instagram followers, “Lil Miquela” is one of the most well-known VIs. She has successfully partnered with the luxury brand Prada for their new collection launch (Yotka, 2018). Further, Lil Miquela endorsed many Samsung products (e.g., Galaxy Z Flip). Through the partnership with a virtual influencer, Samsung was able to convey the futuristic touch of their product successfully (Rasmussen, 2021).

Appel et al. (2020) proposed that VIs could be a potential alternative to actual human influencers. One advantage of using VIs is the reduction of human mistakes in advertising. VIs do not experience anxiety or loneliness when facing uncertainties, enabling them to create posts regularly (Arsenyan and Mirowska, 2021). VIs can also provide advertisers with more control over their influencers’ behavior and content since they are “ageless human robots” and do not have the “offline life” that might potentially impact their online identity (Moustakas et al., 2020). Since VIs exist in the virtual world, there are no constraints, and this allows businesses to be more creative in leveraging VIs, utilizing numerous concepts that human influencers could not possibly handle. However, VIs do have their downsides as they can be perceived as inauthentic or too commercialized. Much like celebrities, brands that use VIs could also suffer from the consequences of endorser transgressions (Louie et al., 2001; Fong and Wyer, 2012; Bartz et al., 2013), as unproven inputs used to post and engage with followers might lead to the spread of disinformation (Thomas and Fowler, 2021).

In this research, we discuss two types of VIs: human-like VIs (HVIs) and anime-like VIs (AVIs). HVIs are digital avatars that are designed to resemble humans, while AVIs appear to be cartoon characters (Arsenyan and Mirowska, 2021).

### 2.2. Consumer response to human-like and anime-like virtual influencers

The CASA paradigm suggests that people unconsciously abide by the same set of social heuristics they use in interpersonal contexts when interacting with computers (Nass and Moon, 2000; Edwards et al., 2019). In other words, people react to computers as they do to humans, especially when there are social heuristics or cues presented (e.g., politeness, humanized voice, and appearances). This resulting anthropomorphism of virtual agents may mitigate uncertainty of interactions and increase perceptions of social presence (Schroeder and Epley, 2016) which increases the likelihood that a human-computer interaction will be similar to human-human ones (Edwards et al., 2019). Some studies that suggest people do react to virtual agents in similar ways as they do to other humans and that regions of the brain that activate during interpersonal experiences so too activate with regard to virtual agents (Kramer et al., 2020).

These days, VIs have more anthropomorphic interfaces and some may look almost indistinguishable from humans in mediated contexts (Lee, 2010). Anthropomorphism applies to perceptions of human attributes in non-human objects which, in turn, may make them seem as though they are capable of social interaction, leading to higher trust (Gong, 2008). Research has shown that people are more likely to trust virtual agents with greater anthropomorphic features and to build a relationship than with their more cartoon-like counterparts due to greater perceptions of social presence (Seymour et al., 2019; Pelau et al., 2021; Liu and Tao, 2022). Additionally, it is thought that media agents such as VIs with higher perceived anthropomorphism may invoke more efficient cognitive processing resulting in a higher likelihood of social interaction (Gambino et al., 2020).

Thus, we expect that VIs, especially those with human-like traits and features to serve as human-like heuristics will lead to people perceiving them as human. In fact, people may feel more connected to HVIs because HVIs offer more social cues, and thus they are more likely to perceive HVI’s endorsements as more credible compared to their counterparts. In contrast, people may find it odd or even suspicious for AVIs to talk about a particular product or brand, as it is more obvious that they are not human.

Regarding the mediating role of message credibility, research has shown that message credibility is an important antecedent of message attitude (e.g., Petty and Cacioppo, 1986; Yoo and Maclnnis, 2005; Xiao et al., 2018; Lee and Kim, 2020). For example, Yoo and Maclnnis (2005) have shown that credibility enhances ad liking and generates favorable ad attitudes. Further, there is ample evidence that ads that lack credibility produce negative emotional and cognitive responses such as disliking the ad and counterarguing (see, e.g., Obermiller et al., 2005). When consumers do not buy into the claims made about a brand endorsed by VIs, their attitudes toward the message will become negative. Therefore, we expect that perceived message credibility will mediate the effect of HVI (vs. AVI) endorsements on message attitudes.

*H1*: HVI (vs. AVI) endorsements will lead to more favorable message attitudes.

*H2*: Message credibility will mediate the effect of HVI (vs. AVI) endorsements on message attitudes.

### 2.3. Virtual influencers and sponsorship disclosure

The relationship between sponsorship disclosure and SMIs has been widely studied in the past (Boerman et al., 2017). FTC guidelines state that when there is any type of financial, work-related, personal, or family connection with the brand, SMIs must disclose sponsorship information (FTC, 2019). These guidelines are meant to alert consumers to advertising content as influencer content is often perceived as more authentic and natural. Though VIs are not currently subject to disclosure rules from the FTC, there is speculation that they will be in the near future (Masteralexis et al., 2021). Many raise a concern about the commercial use of VIs. For example, CNNMoney (2018) argued that “advertisers using computer-generated imagery (CGI) influencers should ensure that the posts are clearly identifiable as advertising.” In this regard, our study will examine the effect of sponsorship disclosure along with the effect of HVI (vs. AVI) endorsements.

Sponsorship disclosure influences the persuasion process and the role of persuasion knowledge within this process because persuasion knowledge may not be activated for consumers who are not aware (Boerman et al., 2012, 2017), it is necessary to inform them that the content is an advertisement through sponsorship disclosure. In general, sponsorship disclosure by influencers has shown to activate defensive and negative attitudes against persuasion attempts, leading to lower ad and brand evaluations (Evans et al., 2017). It has been also suggested that sponsorship disclosure also affects attitude toward advertising and perceived credibility in various contexts. Advertising recognition negatively impacted attitudes toward sponsored posts (Hwang and Jeong, 2016), lowered message credibility, and led to greater scrutiny of the messages (Boerman et al., 2017) due to the material connection of the endorsers with a sponsoring brand/company (De Veirman and Hudders, 2020), resulting in a decrease in the perceived credibility of the endorser.

Therefore, we argue that the negative effect of sponsorship disclosure would hold in the context of virtual influencers as well because disclosed sponsorship should make the VIs’ selling intent obvious to the viewers, resulting in lower message credibility and message attitudes regardless of the type of VIs. In this regard, we are not expecting any significant difference between HVI and AVI endorsements when sponsorship is disclosed.

But the story will be different when there is no sponsorship. People approach social media posts with the presumption that the content is not affected by the products or services mentioned (Carr and Hayes, 2014), but in the case of AVIs, followers can easily infer that such product/brand endorsement is a promotional activity as AVIs are obviously “not real” (Miao et al., 2022). Therefore, we expect HVI (vs. AVI) endorsements lead to more positive message attitudes *via* greater perception of message credibility when sponsorship is not disclosed. However, when the sponsorship is disclosed, the negative effect of disclosure cancels out the superior effect of HVI endorsements, resulting in lower perception of message credibility.

*H3*: Sponsorship disclosure will lead to lower message credibility.

*H4*: The mediating effect of message credibility hypothesized in H2 will be moderated by sponsorship disclosure such that the mediating effect of message credibility will be enhanced when sponsorship is not disclosed rather than disclosed.

## 3. Method

### 3.1. Design, participants, and procedures

Hypotheses were tested in a 2 (VI type: HVI vs. AVI) X 2 (sponsorship disclosure: absent vs. present) between-subjects design. Participants (*N* = 250) were recruited *via* MTurk and were given monetary compensation for their participation. All participants were chosen between 18 and 34 years old based on a recent industry report (Hype Auditor, 2021). Participants who are not active on Instagram or do not drink coffee were initially screened out as our stimuli Instagram post promoted a coffee brand. Further, since we used one of the existing VIs, participants who are familiar with the target VI were screened. Eligible participants were randomly assigned to one of the four study conditions. Approximately 51% of the participants were female, 54% were between 18 and 24 years old, and 72% of them were White.

Participants were told that they would be shown an Instagram post by Jessica (virtual influencer) who is active in the fashion industry with 1 million followers and instructed to view the assigned post as they would view any other social media message. They then responded to the study questions in the following manner: message attitudes, message credibility; an attention-check question; manipulation checks; and demographic questions. In the final analysis, insincere responses (e.g., a uniformed response pattern; *N* = 6) and attention-check failure (*N* = 11) were removed, leaving us 233 usable responses.

### 3.2. Stimuli

The type of VI was manipulated using an animation filter in a photo editing application. We first took a screenshot of the virtual influencer Bermuda’s post from Instagram (@bermudaisbae). Note that we used an existing VI’s photo to enhance ecological validity. As mentioned above, we excluded participants who were familiar with Bermuda from this study. Then, we used a mobile photo application called “Voila” to generate an anime-like version of Bermuda’s face. Additional modifications were made with Adobe Photoshop to ensure that the backgrounds of both images had the same color and texture. For the sponsorship disclosure condition, we added the hashtag #Ad at the beginning of the post. Sample stimuli are shown in Appendix A.

### 3.3. Manipulation checks

The manipulation for VI type was checked with three items taken from Kätsyri et al. (2017) on a 7-point Likert scale (disagree/agree; *M* = 4.20, *SD* = 1.93; *α* = 0.91): “Jessica appeared genuinely human,” “Jessica appeared cartoonish,” and “Jessica’s appearance is exaggerated.” Regarding the sponsorship disclosure manipulation, it was checked with 2 items taken from Boerman et al. (2014) and Darmawan and Huh (2022) on a 7-point Likert scale (disagree/agree; *M* = 4.45, *SD* = 1.46; *r* = 0.69): “The Instagram post I just saw is advertising” and “The Instagram post I just saw contains advertising.”

### 3.4. Measures

Message attitudes were measured with three items taken from Mac Kenzie and Lutz (1989) on a 7-point semantic differential scale: negative/positive, unappealing/appealing, and bad/good (*M* = 4.10, *SD* = 1.31; *α* = 0.90). Message credibility was measured with four items taken from Kim et al. (2017) and Ohanian (1990) on a 7-point Likert scale (disagree/agree): “The Instagram post is generally truthful,” “The Instagram post is believable,” “The Instagram post is deceptive (reverse-coded),” and “The Instagram post leaves on feeling accurately informed” (*M* = 4.181, *SD* = 1.40; *α* = 0.93).

## 4. Results

The manipulation check for VI type was subjected to a 2 (VI type: HVI vs. AVI) X 2 (Sponsorship disclosure: absent vs. present) ANOVA. The results revealed only a significant main effect of the VI type, *F* (1,229) = 1869.50, *p* < 0.001, *η^2^_p_* = 0.91. The AVI condition received significantly higher scores than the HVI condition (*M*_AVI_ = 5.97 vs. *M*_HVI_ = 2.29). A similar ANOVA on the manipulation check for sponsorship disclosure confirmed that the disclosure condition scored significantly higher than the no-disclosure condition on ad recognition (*M*_Disclosure_ = 5.48 vs. *M*_No-disclosure_ = 3.47), *F* (1,229) = 186.64, *p* < 0.001, *η^2^_p_* = 0.48.

**Figure 1:**
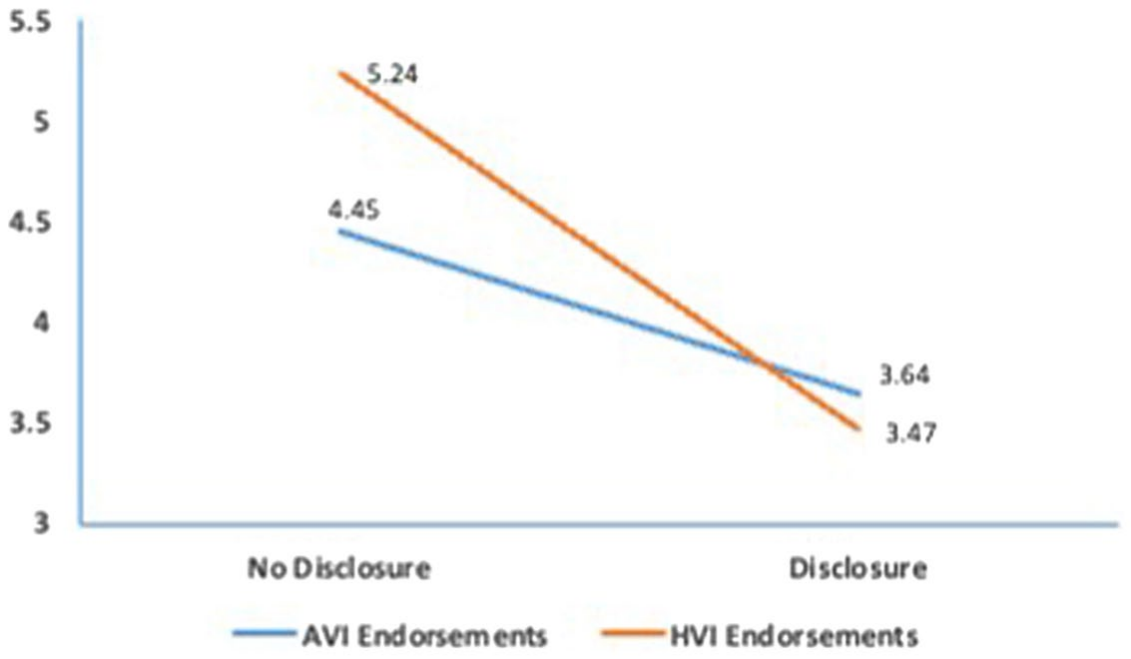
The joint effects of HIV (vs. AVI) endorsements and sponsership disclosure on message credibility.

H1 was tested with a 2 (VI type: HVI vs. AVI) X 2 (sponsorship disclosure: absent vs. present) ANOVA after controlling for age and gender. In support of H1, the results confirmed that HVI endorsements (*M* = 4.26; *SE* = 0.11) produced more positive message attitudes than AVI endorsements (*M* = 3.91; *SE* = 0.11), *F* (1,227) = 4.25, *p* < 0.05, *η^2^_p_* = 0.02. H2 was analyzed in the bootstrapping procedure (10,000 samples) of the PROCESS macro (model 4; Hayes, 2013) after controlling for age and gender. The bootstrap results showed no significant mediation effect of message credibility for the effect of HVI (vs. AVI) endorsements on message attitudes. Thus, H2 was not supported. Regarding H3, the results confirmed a negative effect of sponsorship disclosure on message credibility, *F* (1,227) = 59.37, *p* < 0.001, *η^2^_p_* = 0.24. As predicted, message credibility was lower in the disclosure condition (*M* = 3.54; *SE* = 0.11) than in the non-disclosure condition (*M* = 4.85; *SE* = 0.12). Thus, H3 was supported. Finally, H4 was tested in a moderated mediation model using the bootstrapping procedure (10,000 samples) of the PROCESS macro (model 7; Hayes, 2013) after controlling for the effect of age and gender. In this model, message attitudes served as a dependent variable, VI type as an independent variable, sponsorship disclosure as a moderator, and perceived credibility as a mediator. The bootstrap results confirmed a significant moderated mediation, *B* = −0.58, *SE* = 0.18, 95% bias-corrected CI: −0.97 to −0.23. The direct effect of HVI (vs. AVI) endorsements was not significant, indicating full mediation. Specifically, we found that the mediating effect of perceived credibility for the effect of HVI (vs. AVI) endorsements on message attitudes was significant only when sponsorship was not disclosed, *B* = 0.18, *SE* = 0.09, 95% bias-corrected CI: 0.02 to 0.38. When sponsorship was disclosed, the mediation of message credibility effect became insignificant (the 95% bias-corrected confidence intervals contained zero). A follow-up analysis showed that when sponsorship was not disclosed, HVI endorsements (*M* = 5.24; *SE* = 0.17) resulted in a greater perception of credibility than AVI endorsements (*M* = 4.45; *SE* = 0.17). However, when sponsorship was disclosed, both HVI and AVI endorsements resulted in lower message credibility, and there was no significant difference between the two conditions (*M_HVI_* = 3.47 vs. *M_AVI_* = 3.64). Detailed results are shown in Figure 1. Thus, H4 was supported.

## 5. Discussion

VIs are gaining prominence as a way of attracting people’s attention on social media (Stein et al., 2022), but limited research has been conducted on their effectiveness. Research has mainly focused on the comparison between VIs and human influencers. This exploratory study seeks to provide a deeper understanding on VIs and tests the difference between HVIs and AVIs in terms of their brand endorsement effectiveness. Overall, participants showed more positive message attitudes when they were exposed to HVI endorsements compared to AVI endorsements. Further, Instagram posts with sponsorship disclosure led to a lower perception of message credibility than those with no disclosure. Regarding the moderated mediation, we found that the mediating effect of message credibility is significant only when sponsorship was not disclosed. However, the superior effect of HVIs over AVIs disappeared when sponsorship was disclosed as the message credibility was uniformly low for both conditions, leading to a non-significant mediation.

### 5.1. Theoretical implications

This is one of the first studies to investigate the role of the type of VIs (HVIs vs. AVIs) and sponsorship disclosure plays in consumer responses to their brand endorsement effectiveness, in terms of message credibility and message attitudes. We believe our research advances the prior literature in multiple ways.

First, consistent with previous research (Pelau et al., 2021), our findings confirm the utility of the CASA paradigm to explain the effectiveness of HVI (vs. AVI) endorsements. The CASA paradigm suggests that people react to computers and digital agents as if they were actual social actors. People’s social responses have been noticed to be induced by behavior (Reeves and Nass, 1996) and appearance (Isbister and Nass, 2000) of the agent. The extended CASA paradigm by Gambino et al. (2020) also proposes that how people interpret the social potential of the media agent relates to social affordances. The technological advances in CGI and AI have broadened the scope of communicative cues, hence virtual agents that display more human-like cues have been received more positively (Gambino et al., 2020).

Second, this study further supports the proposition of previous research (Hwang and Jeong, 2016) on the negative effects of sponsorship disclosure. The notice of sponsorship disclosure causes social media users to perceive the content as advertising (Evans et al., 2017). The hashtag “#Ad” activates the persuasion knowledge (Wojdynski and Evans, 2016), which decreases the message credibility of the VI as the endorser’s ulterior intentions (e.g., monetary compensation) become apparent. This finding is also in line with research on SMI sponsorship based on persuasion knowledge (Boerman et al., 2012, 2017). When followers notice a sponsorship disclosure, they connect the VI’s favorable endorsement toward the product to the compensated relationship between the endorser and the product rather than the VI’s positive recommendation about the product, leading followers to devalue the credibility of the message.

Third, our results show that the importance of message credibility as a crucial precursor for promoting positive attitudes is carried over to the context of VI advertising as well. Past studies have demonstrated that perceived credibility is essential for SMIs in encouraging followers to develop positive attitudes (Belanche et al., 2021). The VI type (human-like vs. anime-like) and sponsorship disclosure interacted in a way that messages from HVIs with no sponsorship alert led to higher credibility. Therefore, it should be acknowledged that the effectiveness of influencer marketing does not rely only on the message of the post itself but is also affected by the type of SMIs.

### 5.2. Managerial implications

This study also provides useful insights for marketing and social media professionals by showing that VIs can be a viable alternative to endorsing celebrities or SMIs. First, it is recommended that marketers may want to use HVIs over AVIs since participants have more favorable attitudes toward HVIs than AVIs. The disparity in message attitude and credibility between the HVI and AVI, despite having the same content and format, exemplifies the need to maximize the realism of VI characters to make the audience believe they genuinely exist.

Our findings show that sponsorship disclosure elicits negative responses from participants. Advertisers should carefully consider solutions to minimize the negative effect caused by sponsorship disclosure. For example, managers may seek contextual fit with a virtual influencer which may mitigate the ding to credibility increased by a sponsorship disclosure (see: Schouten et al., 2020). Additionally, companies should work to increase perceived transparency not only that they are virtual but also about their partnerships with brands as a way to develop trust (Moustakas et al., 2020).

### 5.3. Limitations and future research

Although our research offers exciting insights on the VIs and brand sponsorship, a few limitations and future research suggestions are worth noting. First, being exploratory in nature, this research focused on the effect of HVIs (vs. AVIs). Given that there are various types of VIs such as anime-like (e.g., Noonoouri), animal-shaped (e.g., Geico), and food-shaped (e.g., Nobody Sausage), it will be interesting to see if our findings can be extended to those non-human VIs. Future research could test our findings with a different product category (e.g., beauty and fashion) too. Additionally, this research focused on participants from 18 to 34 years old, as they are reported as the primary audience of VIs on Instagram (Hype Auditor, 2021). Future research may want to validate our findings with a broader participant pool by including younger (e.g., Gen Alpha) and/or older age (e.g., Gen X) groups. Lastly, it is important to keep in mind that research around VIs is still in its early stage. As people become more familiar with the concept of VIs, people’s reactions might evolve.

## Data availability statement

The raw data supporting the conclusions of this article will be made available by the authors, without undue reservation.

## Ethics statement

The studies involving human participants were reviewed and approved by USC Institutional Review Board, University of Southern California. The patients/participants provided their written informed consent to participate in this study.

## Author contributions

EK: study conception and design, data collection and analysis, final manuscript writing, project administration and supervision, and funding acquisition. DK: study conception, manuscript draft preparation, final manuscript writing, and stimulus development. ZE: study conception, stimulus development, and final manuscript writing. HS: final manuscript writing and editing. All authors contributed to the article and approved the submitted version.

## Conflict of interest

The authors declare that the research was conducted in the absence of any commercial or financial relationships that could be construed as a potential conflict of interest.

## Publisher’s note

All claims expressed in this article are solely those of the authors and do not necessarily represent those of their affiliated organizations, or those of the publisher, the editors and the reviewers. Any product that may be evaluated in this article, or claim that may be made by its manufacturer, is not guaranteed or endorsed by the publisher.

## Supplementary material

The Supplementary material for this article can be found online at: https://www.frontiersin.org/articles/10.3389/fpsyg.2023.1089051/full#supplementary-material
