# Supplementary material for: The next hype in social media advertising: Examining virtual influencers’ brand endorsement effectiveness
Source: Front Psychol. 2023 Feb 24;14:1089051. doi: 10.3389/fpsyg.2023.1089051 (PMC10026852; doi:10.3389/fpsyg.2023.1089051)

**Appendix A: Sample Stimulus – A mock-up of the actual stimuli including some text from the HVI and sponsorship disclosure condition**


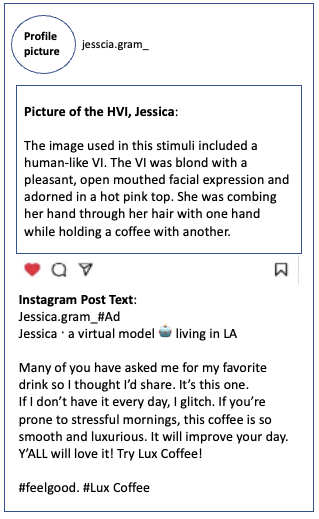


**Sample Stimulus – A mock-up of the actual stimuli including some text from the AVI and sponsorship disclosure condition**


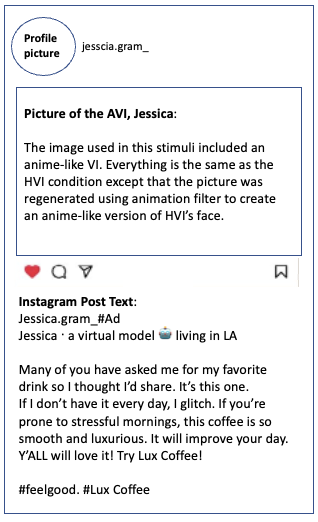

Supplement: Supplementary file 1 [file Data_Sheet_1.docx]
